# Metabolic Syndrome in Arab Adults with Low Bone Mineral Density

**DOI:** 10.3390/nu11061405

**Published:** 2019-06-21

**Authors:** Kaiser Wani, Sobhy M. Yakout, Mohammed Ghouse Ahmed Ansari, Shaun Sabico, Syed Danish Hussain, Majed S. Alokail, Eman Sheshah, Naji J. Aljohani, Yousef Al-Saleh, Jean-Yves Reginster, Nasser M. Al-Daghri

**Affiliations:** 1 Chair for Biomarkers of Chronic Diseases, Department of Biochemistry, College of Science, King Saud University, Riyadh 11451, Saudi Arabia; wani.kaiser@gmail.com (K.W.); sobhy.yakout@gmail.com (S.M.Y.); ansari.bio1@gmail.com (M.G.A.A.); eaglescout01@yahoo.com (S.S.); danishhussain121@gmail.com (S.D.H.); msa85@yahoo.co.uk (M.S.A.); alaslawi@hotmail.com (Y.A.-S.); jyr.ch@bluewin.ch (J.-Y.R.); 2 Diabetes Care Center, King Salman Bin Abdulaziz Hospital, Riyadh 12769, Saudi Arabia; eman_shesha@hotmail.com; 3 Specialized Diabetes and Endocrine Center, King Fahad Medical City, Riyadh 12231, Saudi Arabia; najij@hotmail.com; 4 College of Medicine, King Saud bin Abdulaziz University for Health Sciences, Riyadh 22490, Saudi Arabia; 5 King Abdullah International Medical Research Center, Riyadh 11481, Saudi Arabia; 6 Department of Medicine, Ministry of the National Guard—Health Affairs, Riyadh 14611, Saudi Arabia; 7 Department of Public Health, Epidemiology and Health Economics, University of Liège, 4000 Liège, Belgium

**Keywords:** metabolic syndrome, bone mineral density, obesity, insulin resistance, bone health, osteoporosis

## Abstract

There are discrepancies in the reports on the association of metabolic syndrome (MetS) and its components with bone mineral density (BMD) and hence more population-based studies on this subject are needed. In this context, this observational study was aimed to investigate the association between T-scores of BMD at lumbar L1–L4 and full MetS and its individual components. A total of 1587 participants (84.7% females), >35 years and with risk factors associated with bone loss were recruited from February 2013 to August 2016. BMD was done at L1–L4 using dual-energy X-ray absorptiometry (DXA). T-Scores were calculated. Fasting blood samples and anthropometrics were done at recruitment. Fasting lipid profile and glucose were measured. Screening for full MetS and its components was done according to the National Cholesterol Education Programme Adult Treatment Panel III (NCEP ATP III) criteria. Logistic regression analysis revealed that the odds of having full MetS increased significantly from the lowest T-score tertile to the highest one in both sexes (OR, odd ratio (95% CI, confidence interval) of tertile 2 and 3 at 1.49 (0.8 to 2.8) and 2.46 (1.3 to 4.7), *p* = 0.02 in males and 1.35 (1.0 to 1.7) and 1.45 (1.1 to1.9), *p* < 0.01 in females). The odds remained significant even after adjustments with age, body mass index (BMI), and other risk factors associated with bone loss. Among the components of MetS, only central obesity showed a significant positive association with T-score. The study suggests a significant positive association of T-score (spine) with full MetS irrespective of sex, and among the components of MetS this positive association was seen specifically with central obesity.

## 1. Introduction

Metabolic syndrome (MetS), a syndrome consisting of several disorders like abdominal obesity, dyslipidemia, increased blood pressure and impaired glucose regulation, represents characteristics such as low-grade inflammation and increased oxidative stress [1,2]. The increased prevalence of MetS in Saudi Arabia in recent years is blamed on rapid economic growth and Westernization of lifestyle [3,4]. The clinical impact of MetS is immense, considering its vascular harm and its predisposition to the progression of cardiovascular diseases and metabolic complications like type 2 diabetes mellitus (T2DM) [5,6].

The National Cholesterol Education Programme Adult Treatment Panel III (NCEP ATP III) [7] definition for full MetS requires the presence of at least three out of five components namely central obesity, hyperglycemia, low high-density cholesterol, hypertriglyceridemia and hypertension. The NCEP-ATPIII definition of MetS is the most commonly used definition for epidemiologic studies in the region since it does not require a pre-requisite risk factor (e.g., obesity for the International Diabetes Federation (IDF), hyperglycemia for the World Health Organization (WHO)) to reach a diagnosis. Interestingly, these components of MetS may also affect the bone mineral density (BMD) and bone metabolism through several mechanisms, one of which is the reduced blood flow to bone mass due to micro-vascular complications associated with impaired glucose regulation [8]. The other proposed mechanism is hypercalciuria induced by higher glucose levels and elevated blood pressure [9]. In spite of the associations, the overall relationship of MetS components, bone health and BMD remains blurred. For instance, although individuals with T2DM are at an increased risk for fractures [10], higher BMDs are seen in T2DM individuals [11,12]. However, a recent meta-analysis conducted on eight epidemiologic studies involving 39,938 participants suggested no explicit effect of MetS on bone fractures [13]. Apart from this, there are discrepancies in the reports on the association of MetS components and bone health in different sexes [14,15].

Clearly, more population-based studies are needed to decipher the relationship between MetS and its components on bone health. The authors’ hypothesize a statistically significant association between different components of MetS and full MetS with the BMD at lumbar L1–L4 bone site. In this context, this observational study was aimed to investigate the association between T-scores of BMD at lumbar L1–L4 and full MetS and its individual components in Saudi Arab adults with risk factors associated with bone loss. Both sexes were included in this study to investigate the sexual disparity, if any, in these associations.

## 2. Materials and Methods

This observation study is part of a larger study dedicated to finding the biomarkers associated with bone health (titled: Osteoporosis Disease Registry); it was approved by the Ethics Committee at College of Science, King Saud University (KSU), Saudi Arabia (# H-01-R-012). 

### 2.1. Study Design and Participants

The study was conducted in several hospitals around Riyadh city; the most prominent of them being King Fahad Medical City (KFMC), King Khalid University Hospital (KKUH) and King Salman Hospital (KSH), where the bulk of the recruitment was done. The study began in early 2013 and the recruitment ended in August 2016. The inclusion criteria were consenting males and females, >35 years old, who in their general physician visits had been advised of a bone mineral density (BMD) scan owing to their being at a risk associated with bone loss. There were no specific exclusion criteria except being ≤35 years of age or participants with malignancy, cardiac or lung diseases, etc. that required immediate medical attention. A total of 1587 participants (84.7% females and 15.3% males) were recruited during the period.

### 2.2. Bone Mineral Density (BMD) Scan and T-Score

A dual-energy X-ray absorptiometry (DXA) machine (Hologic Inc., Marlborough, MA, USA) was utilized to measure the bone mineral density (BMD) at lumbar vertebrae L1–L4 and the average scores were used to calculate sex-specific T-scores by the software installed in the machine which shows how much the measured bone density is higher or lower than the bone density of a healthy 30-year-old same sex individual. The machine was calibrated using a standard phantom provided by the manufacturer and the test was performed by a certified bone densitometry technologist. The results of the bone density scan were printed and transported to the Chair for Biomarkers in Chronic Diseases (CBCD) at KSU for data entry. 

### 2.3. Anthropometry, Blood Collection and Sample Analysis

The study participants were invited for a fasting blood withdrawal procedure and administration of a standard questionnaire on the risk factors associated with the bone loss. Anthropometry measurements included height, weight, waist and hip circumference, and systolic/diastolic blood pressure, measured by trained nurses using standard procedures. Weight (Kg) was recorded using an international standard scale (Digital Pearson scale, ADAM Equipment Inc., Oxford, CT, USA). Height and waist and hip circumference (to the nearest 0.5 cm) were measured utilizing a standardized measuring tape. Body mass index (BMI) was calculated as weight in Kgs divided by height in square meters. Blood pressure (mmHG) was recorded by a trained nurse twice after 10 minutes rest using a conventional mercurial sphygmomanometer and the average was noted. A one-on-one interview was conducted by trained research associates where the participants were informed about the study and reported risk factors associated with bone loss through a 12-point questionnaire. The risk factors included whether or not the subject had T2DM, thyroid dysfunction, family history of diabetes, osteoporosis, arthritis, etc., whether they had a barium test, presence of scoliosis or kyphosis, loss of height in the last two years and history of fractures in the last five years.

Collected fasting blood samples were transported to CBCD laboratory for biochemical analysis which included lipid profile as well as glucose, calcium and albumin analysis quantified using an automated biochemical analyzer (Konelab 20, Thermo-Fischer Scientific, Espoo, Finland). The reagents were purchased from Thermo Fischer (catalogue# 981379 for glucose, 981812 for total cholesterol, 981823 for high density lipoprotein (HDL)-cholesterol, 981301 for triglyceride, 981367 for calcium and 981766 for albumin). Glucose kit employed the routine glucose-oxidase, peroxidase method; total cholesterol test employed cholesterol-esterase, oxidase method; HDL-cholesterol test employed the two-step polyethylene glycol modified cholesterol esterase and oxidase methodology; triglyceride kit employed enzymes like lipoprotein lipase and oxidase; calcium kit employed Arsenazo III methodology while albumin kit employed bromocresol purple method. The imprecision calculated as the total coefficient of variation (CV) was ≤5%, ≤3.5%, ≤4%, ≤4%, ≤4.5% and ≤3% for these tests, respectively. Insulin was also measured in these samples using Luminex multiplex (Luminexcorp, Austin, TX, USA) with fluorescent microbead technology.

### 2.4. T-Score Tertiles and MetS Components

Data for both males and females were divided into T-score tertiles with tertile 1 having the lowest T-score (spine) and tertile 3 having the highest T-score. The status of full MetS and its five components was assessed as present/absent (dichotomous data) using the criteria set in the National Cholesterol Education Programme Adult Treatment Panel III (NCEP-ATP III) [16] which states MetS being present if at least three of the following five components are present.

1. Central obesity: waist circumference >102 cm (males), >88 cm (females).
2. Hyperglycemia: fasting glucose >5.6 mmol/l or pharmacologic treatment for hyperglycemia (both sexes).
3. Hypertriglyceridemia: serum triglycerides ≥1.7 mmol/l (both sexes).
4. Low HDL-cholesterol: serum HDL-cholesterol <1.03 mmol/l (males), <1.30 mmol/l (females).
5. Hypertension: systolic blood pressure >130 mmHg and/or diastolic blood pressure >85 mmHg or current use of antihypertensive medications.

### 2.5. Data Analysis

SPSS (Version 23.0, SPSS Inc., Chicago, IL, USA) was used to analyze the data. Kolmogorov–Smirnov test was employed to assess the normal distribution of the preliminary data. Continuous normally distributed variables were presented as mean ± standard deviation while median (25th and 75th percentile) was used for continuous non-normal variables. Categorical variables like status of full MetS and its individual components; and data obtained from the bone loss risk factor questionnaires were presented as percentages. Chi-squared test (3 × 2 contingency table) was used for checking the differences in prevalence of MetS and its components in the three tertiles of T-scores. Multinomial logistic regression was performed using the T-score tertile as a dependent variable (with lowest tertile as reference) and full MetS or its individual components (present vs. absent) as independent variables. Data was presented as odds ratio 95% confidence interval (OR (95% CI)) and respective *p*-values represented odds of having different components of MetS at higher tertiles of T-score compared to the lowest. Different models were employed with model “a” as univariate, and all other models were adjusted accordingly for + age (model “b”), + BMI (model “c”), + other components of MetS (model “d”) and + risk factors associated with bone loss like T2DM, thyroid dysfunction, etc.; family history of diabetes, osteoporosis, arthritis, etc.; barium test done previously; scoliosis or kyphosis of spine; loss of height reported; history of broken bones (model “e”). The analysis was conducted at 95% confidence level and a *p*-value < 0.05 was considered statistically significant. MS excel 2010 was used to prepare figures.

## 3. Results

### 3.1. General and Biochemical Characteristics of the Study Participants

Table 1 show the general and biochemical characteristics of the study participants. A total of 1587 participants were recruited for the study, out of which 84.7% (*N* = 1344) were females. Participants who were older than 35 years with risk factors associated with bone loss were recruited. The mean age for females and males were 56 and 58 years, respectively. It was determined that 87.3% of females and 79.4% of males were either overweight or obese (BMI >25 kg/m^2^) with an average BMI in females at 32.93 kg/m^2^ and males at 29.86 kg/m^2^. Among the risk factors associated with bone loss, the most prominent in the participants were age greater than 50 years (77% in females and 79.8% in males); family history of diabetes (58.6% in females and 74.5% in males); and those diagnosed with T2DM (51.4% in females and 70.8% in males). The other risk factors like family history of osteoporosis and arthritis were moderately found in both males and females. The percentage of females who reported having thyroid disease, done a barium test (last two weeks of recruitment), and had scoliosis of the spine was 9.2%, 10.7% and 8.9%, respectively, while these risk factors were negligibly found in males. Additionally, 10% and 11.7% of the females reported loss of height in the last two years and a history of broken bones in the last five years of recruitment, respectively. The median (Q1, Q3) values for T-score at spine (L1–L4) for females and males were –1.70 (−2.5, −0.8) and −0.91 (−1.7, 0.1), respectively. The table also shows the biochemical characteristics of the participants, like lipid profile and fasting glucose, that were analyzed to assess different components of MetS. 

### 3.2. Prevalence of Different MetS Components in T-Score Tertiles

Table 2 shows the prevalence of the five components of MetS and full MetS in participants with tertiles according to the T-score (L1-L4 spine). The lowest T-score (least BMD) in both groups represents the tertile 1 and the highest T-score represents the tertile 3. Among the five components of MetS, the prevalence of central obesity was significantly different in the three T-score tertiles in all participants as well as in males and females. It increased from 66.9% in tertile 1 to 82.7% in tertile 3 (*p* < 0.01). A similar trend was followed in males where it increased from 38.3% in tertile 1 to 71.6% in tertile 3 (*p* < 0.01), and in females where it increased from 72.1% in tertile 1 to 84.7% in tertile 3 (*p* < 0.01). The prevalence of other four components (hyperglycemia, low HDL-cholesterol, hypertriglyceridemia and hypertension) was more or less similar in different tertiles of T-score and this was seen in both males and females except for hypertriglyceridemia in all participants where it increased from 40.3% in tertile 1 to 47.7% in tertile 3 (*p* = 0.04). The prevalence of full MetS, however, increased significantly from the lowest T-score tertile to the highest and this was seen in all participants as well as when data was divided according to sexes (56.1% in tertile 1 to 67.7% in tertile 3, *p* < 0.01 in all participants as well as in males where full Mets was 50.6% in tertile 1 and 71.6% in tertile 3, *p* = 0.02; and females where it increased from 57.1% to 65.8%, *p* = 0.003).

**Figure 1:**
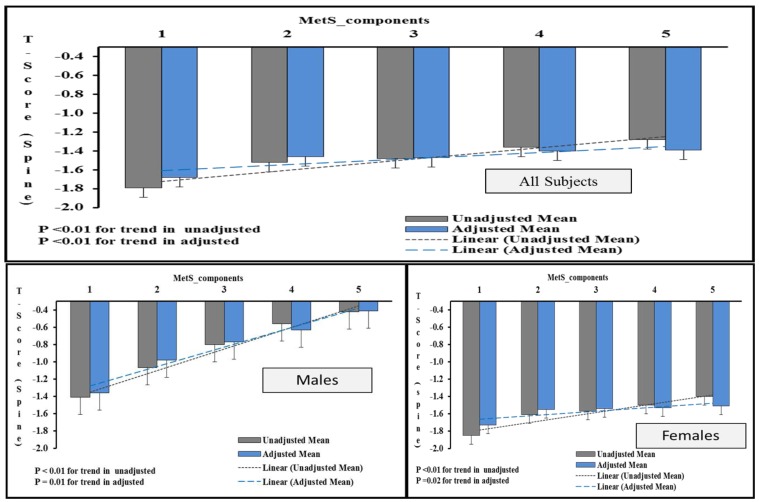
Increasing T-score (spine) values with increasing MetS components according to sex. Note: The figure shows the average T-score (spine) in participants with 1 or more MetS components. The data was generated by univariate analysis by taking T-score (spine) values as dependent variables and “number of components” as factors. The values were adjusted for age, BMI and other risk factors associated with bone loss. *p* < 0.05 was considered as significant.

Figure 1 shows the average T-score (spine) in individuals with one or more MetS components in males and females. A statistically significant trend was observed in both sexes with average T-score values increasing with increasing MetS components and this remained so even after multiple adjustments.

### 3.3. Association of T-score with Different Components of MetS and Full MetS.

Table 3 shows the results of a multinomial regression analysis done using T-score (spine) tertiles as dependent variables (with lowest tertile as reference) and different components of MetS as factors. The results are shown as odds ratio (95% confidence interval) and respective *p*-values representing odds of having different components of metabolic syndrome with different tertiles of T-score. The odds of having full MetS increased significantly from lowest tertile to the highest tertile of T-score in all participants (OR (95% CI) of tertile 2 and 3 at 1.55 (1.2 to 2.0) and 1.57 (1.2 to 2.0), *p* (trend) < 0.01 as well as in both males and females (1.49 (0.8 to 2.8) and 2.46 (1.3 to 4.7), *p* (trend) = 0.02 in males and 1.35 (1.0 to 1.7) and 1.45 (1.1 to 1.9), *p* < 0.01 in females) and the odds ratio remained statistically significant even after multiple adjustments with age, BMI and other risk factors associated with bone loss. Among the components of MetS, the odds of hyperglycemia and hypertriglyceridemia increased significantly with T-score tertiles when unadjusted only in all participants, however, the association lost significance after adjusting with confounders. This association was not significant when the data was divided according to sexes. Only the odds of central obesity as a component of MetS increased significantly with T-score tertiles in univariate as well as adjusted models, and this trend was seen when investigated in all participants as well as when the data were divided into different sexes.

**Figure 2:**
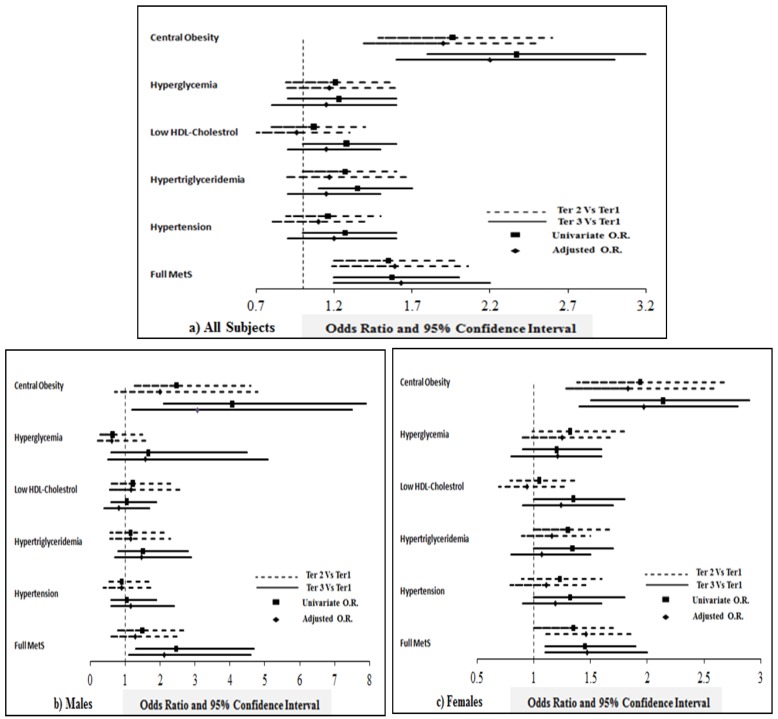
Odds of having full MetS and its components in individuals with higher tertiles of T-score (spine) compared with lowest tertile. Note: The figure shows the odds ratio (OR) and 95% confidence interval representing odds of having different components of metabolic syndrome at higher tertiles of T-score (spine) compared to the lowest tertile. The data was generated by multinomial regression taking T-score tertiles as dependent variables and MetS and its components (present versus absent) as factors. Unadjusted OR; and OR adjusted for age, BMI and risk factors associated with bone loss like family history of diabetes, osteoporosis, arthritis, whether or not suffering from T2DM, thyroid disease, history of broken bones, etc., are represented here.

Figure 2 shows the odds ratio and its associated 95% confidence interval of different components of MetS in higher tertiles of T-score (spine) compared with the lowest tertile.

## 4. Discussion

The current population-based study aimed to investigate the relationship between MetS and its components with the BMD of the spine. The main novelty of the study is that it is the first large-scale investigation on the association of MetS and BMD in a homogenous Arab ethnic group. This is clinically relevant since it is well established that bone health and risk for certain chronic conditions are influenced by genetics and environment. Several inconsistencies in associations have been found in other studies and one of the main reasons is the differences in population. The study fills this gap as it has never been investigated in this region; a well-structured logistic regression model with multiple adjustments for risk factors associated with bone loss differentiates this study from others on a similar topic. In this study, the authors found a significant positive association of full MetS with BMD spine and this association was independent of sex. The odds ratio of having full MetS, as revealed by logistic regression analysis, increased significantly from the lowest T-score tertile to the highest one and the odds remained significant even after adjustment with multiple confounders and the trend revealed similar association across both sexes. This significant positive association of MetS with BMD seemed to be driven by central obesity as all other components of MetS showed little or no association with increasing tertiles of T-score. 

Many recent studies in varied populations were conducted to investigate the associations between MetS, BMD, osteoporosis and fractures. In these studies, the difference in MetS selection criteria, difference in the bone site used to calculate BMD like femoral neck, lumbar spine etc., or the difference in osteoporotic fractures and non-vertebral fractures, played a role in these associations, yet the overall picture of the relationship remains blurred. In one of the earliest studies by Muhlen et al. [17], significantly lower BMD at total hip was associated with MetS in age-adjusted models. Also, in a report by Hwang et al. [18], vertebral BMD was found to be significantly lower in women with MetS compared to non-MetS. Similarly, Jeon et al. [19] suggested higher BMD at the lumbar spine and femoral neck in post-menopausal women with MetS. The results from these studies are in conflict with the present study where a significant positive association was seen between MetS and lumbar BMD. The difference might be because of the lower prevalence of MetS in these studies [17,18,19] (20.9%, 20.7% and 9.0%, respectively) than the present study (62.3%), suggesting that the participants recruited in these studies were healthier than the current study group. Also, the study participants used by Muhlen et al. were much older than our study (mean age of 74.2 and 74.4 years in men and women, respectively, compared to 58.1 and 56.4 years in this study). The results in the current study are in line with some of the earlier reports [20,21,22] which suggest women with MetS have a significantly higher BMD than those without MetS. A meta-analysis of a total of 11 studies including 13,122 participants [23] revealed a significant overall association between MetS and increased BMD of spine, in concordance with the present study results.

The present study results of the positive association of full MetS with BMD spine seem to be driven by the component of central obesity as only this component predominantly follows the same pattern in its relationship with BMD spine as the full MetS (Table 3). A positive association of central obesity with BMD at all sites was reported first by Edelstein et al. [24] and since then BMI has been reported as a protective factor against bone loss as concluded by a meta-analysis of 12 studies including 60,000 participants [9]. However, as a flipside to this story, some studies advocate that increased obesity is correlated to low bone mass [25,26]. This paradox in the nature of this relationship of obesity with bone health may be explained by the heterogeneous role of obesity. On one hand higher waist circumference used to calculate central obesity as a component of MetS is associated with higher 17 beta-estradiol levels which may protect bone [27], while on the other hand, intra-abdominal fat or visceral fat may lead to increased bone resorption and hence lower BMD because of its association with pro-inflammatory cytokines [28,29]. Heavy weight is indeed one of the consequences since it is well-established that increased mechanical loading conferred by body weight (e.g., increased BMI or being overweight/obese) has a positive effect on bone formation, regardless of other consequences such as increased risk for other chronic health disorders [30].

The association of other components of MetS with BMD, like full MetS, as reported by many studies in the recent past is also inconclusive. In our study, in all participants, hyperglycemia increased significantly with increasing tertiles of T-score in an unadjusted model but lost significance after adjustment with multiple confounders (Table 3). This observation is supported by some of the earlier reports [31,32] which suggest an indirect effect of hyperglycemia through associated hyperinsulinemia on bone formation, as interaction between insulin, insulin like growth factor-1 and parathyroid hormone has been proposed to have an anabolic effect on bone cells [33]. At the same time, however, long-term diabetes has consistently been shown to be detrimental for bone health and associated fractures [10,34]. A direct effect of hyperglycemia and hyperinsulinemia on bone health and associated fractures would be interesting to investigate. Also, hypertriglyceridemia in our study increased, in all participants, with increased tertiles of T-score in an unadjusted model but lost significance after adjustment. Elevated triglycerides have been suggested to contribute to the overall improvement of qualitative properties of bone by forming a layer between collagen fibers and mineral crystals [35]. We did not observe a significant relationship between other components of MetS and T-score spine, but the results of some other studies present a conflicting picture [36,37]. 

In our study, no sexual disparity was observed as far as the relationship of MetS and T-score spine was concerned. In both sexes, a positive correlation was found in this relationship and the odds of having MetS increased significantly with higher tertiles of T-score spine even after multiple adjustments. This relationship seems to be driven by the central obesity component in both sexes. Similar to our observations, a meta-analysis conducted on participants showed no gender differences [23]. In contrast, some studies suggest gender disparity in the relationship between MetS and BMI-adjusted BMD, showing a less beneficial effect in men than women [15]. It is difficult to explain this disparity in different studies; however, an explanation might be in the age group of the study participants (e.g., the study that suggests gender differences in the relationship between MetS and BMD was conducted in much younger participants than ours (mean age 56.7 years) and it has been shown that BMD overall decreases as age progresses and the rate of decline in the BMD differs according to sex) [38].

The heterogeneity of the complex relationship between MetS and BMD as seen in various studies before may be explained on the basis of various study-level variables like ethnicity, DXA scanner manufacturer and MetS definition [23]. MetS in Asians may be more influenced by visceral adiposity as compared to Caucasians [39] and hence may explain the positive relationship of MetS and BMD seen in Caucasians rather than Asians. The choice of the DXA scanner used may also play a role in the relationship as the absolute values of BMD differ between instruments from different manufacturers [40]. Also, the differences in the definition of the MetS used in various such studies add to the heterogeneous relationship between MetS and BMD. Abdominal obesity is a necessary factor for the International Diabetes Federation (IDF) criteria of MetS whereas NCEP-ATP III criteria categorizes MetS by having any three of the five components. This difference in MetS definition may dictate the observable positive association of MetS defined by NCEP-ATP III as seen in many studies before including ours rather than when MetS was defined by IDF criteria.

Higher waist circumference (central obesity), is associated with higher 17 β-estradiol levels which is known to improve bone density by inhibiting bone resorption and stimulating bone formation [27]. On the other hand, triglycerides affect osteoblast and osteoclast differentiation, with lower levels associated with osteoporotic bone tissue [41]. Lastly, hyperglycemia and hyperinsulinemia have been demonstrated to exert an anabolic effect on bone cells by interacting insulin-growth factor (IGF)-1 receptors which are known to increase BMD by promoting osteoblast differentiation [42]. It is worthy to note that higher BMD alone cannot account for improved fracture risk since patients with T2DM and those who are obese are still at an increased risk for fragility fractures and associated mortality [43,44]. MetS being a constellation of interconnected physiological, biochemical, clinical and metabolic factors has in itself two contrasting factors: one of which is abdominal obesity known to be protective against osteoporosis and may lead to higher BMD levels [45,46] while at the same time intra-abdominal obesity, hyperglycemia and insulin resistance leads to a low-grade inflammatory state that activates bone resorption [28,47]. This might explain this heterogeneous complex relationship, however, more such studies need to be conducted in different populations to fully elucidate the pathophysiology behind this complexity. 

There are several strengths of the current study. First, the sizable study sample of 1587 participants gives this study an edge over many other studies with fewer participants. Secondly, the multi-center recruitment of the study participants used in this study provides a proper representation of a population. Thirdly, this is the first study to report the relationship of MetS with BMD spine in a population with high prevalence of MetS as well as osteoporosis. Apart from these, the use of the most recent definition of MetS, well-calibrated DXA scans performed by a certified bone densitometry technologist and a well-structured logistic regression model analysis adjusted by multiple confounders gave reliable results. The authors, however, acknowledge certain limitations. The cross-sectional nature of this study limits its applicability outside cause and effect of the proposed relationship. A longitudinal study in this population would be interesting to investigate. Some of the biochemical markers that may influence BMD like 25(OH) vitamin D, serum estradiol, follicle stimulating hormone, etc. were not measured in this study. Also, the BMD was measured only at lumbar L–L4 site and hence the proposed relationship with MetS may not hold true for other anatomic sites like femoral neck, total hip, etc.

## 5. Conclusions

The results of the logistic regression analysis with multiple adjustments conducted in this study suggest a sex-irrespective positive association between MetS and lumbar BMD. The results also suggest that the positive relationship between MetS and lumbar BMD is predominantly driven by the central obesity component of MetS as the rest of the components show little or no association with lumbar BMD, irrespective of sex. 

## Figures and Tables

**Table 1:** General and biochemical characteristics of participants.

	Overall	Males	Females
*N*	1587	243	1344
Anthropometrics			
Age (years)	56.7 ± 8.2	58.1 ± 9.4	56.4 ± 7.9
BMI (kg/m^2^)	32.4 ± 6.4	29.9 ± 5.2	32.93 ± 6.4
Waist (cm)	100.3 ± 14.1	103.7 ± 14.2	99.7 ± 14
Hips (cm)	107.9 ± 12.9	104.4 ± 12	108.6 ± 13
Systolic BP (mmHg)	126.2 ± 16.4	131.1 ± 13.1	125.4 ± 16.8
Diastolic BP (mmHg)	76.8 ± 9.9	79.5 ± 8.2	76.3 ± 10.1
Risk Factors			
Age (>50 years) ^$^	77.4	79.8	77.0
Family History			
Diabetes Mellitus ^$^	61.0	74.5	58.6
Osteoporosis ^$^	9.8	9.9	9.7
Arthritis ^$^	6.2	4.9	6.5
Subject History			
Diabetes Mellitus ^$^	54.4	70.8	51.4
Thyroid Disease ^$^	8.1	2.5	9.2
Barium Test (last 2 weeks) ^$^	9.4	2.1	10.7
Scoliosis of Spine ^$^	7.8	1.6	8.9
Kyphosis ^$^	3.5	0.8	4.0
Lost Height (2 years)^ $^	8.8	1.6	10.0
Fracture (last 5 years)^ $^	10.7	5.3	11.7
T-Score (L1–L4 Spine)	–1.49 ± 1.3	–0.84 ± 1.3	–1.60 ± 1.3
Biochemical Characteristics			
Glucose (mmol/l) ^#^	6.7 (5.5, 9.9)	9.2 (6.5–14.1)	6.5 (5.4–9.3)
Insulin (μU/ml)^ #^	10.4 (5.9, 18.4)	21.6 (10.7, 33.6)	9.4 (5.4, 15.6)
Total Cholesterol (mmol/l)	5.0 ± 1.1	4.9 ± 1.3	5.08 ± 1.1
Triglycerides (mmol/l) ^#^	1.6 (1.2, 2.2)	1.9 (1.4, 2.8)	1.55 (1.1, 2.2)
HDL-Cholesterol (mol/l)	1.2 ± 0.4	1.1 ± 0.3	1.17 ± 0.4
Calcium (mmol/l)	2.3 ± 0.3	2.3 ± 0.2	2.3 ± 0.3
Albumin (g/l)	38.7 ± 6.7	40.2 ± 5.8	38.4 ± 6.8

**Note:** Data presented as mean ± standard deviation for normal variables; median (Q1, Q3) for non-normal variables (#) and as frequency (%) for categorical variables ($). BMI, body mass index; BP, blood pressure.

**Table 2:** Prevalence of metabolic syndrome (MetS) components in tertiles of T-score (spine) according to sex.

	All (*N* = 1587)				Males (*N* = 243)				Females (*N *= 1344)			
T-Score (L1–L4 Spine)	T1	T2	T3	*p*	T1	T2	T3	*p*	T1	T2	T3	*p*
Central Obesity	66.9	79.8	82.7	<0.01	38.3	60.5	71.6	<0.01	72.1	83.4	84.7	<0.01
Hyperglycemia	69.9	73.8	74.0	0.25	86.4	80.2	91.4	0.12	66.9	72.7	70.8	0.16
Low HDL-Cholesterol	63.0	64.6	68.5	0.17	40.7	45.7	42.0	0.80	67.1	68.1	73.3	0.09
Hypertriglyceridemia	40.3	46.2	47.7	0.04	54.3	58.0	64.2	0.43	37.7	44.0	44.6	0.07
Hypertension	29.3	32.5	34.4	0.20	38.3	35.8	39.5	0.88	27.6	31.9	33.5	0.15
Full MetS	56.1	63.3	67.6	<0.01	50.6	60.5	71.6	0.02	57.1	64.4	65.8	0.003

Note: Data presented as frequency (%) for the components of MetS and full MetS. T1, T2 and T3 are the three tertiles of T-score (spine) whose respective values as median (Q1, Q3) are −2.70 (−3.1, −2.4), −1.65 (−1.9, −1.3) and –0.30 (−0.7, 0.4) for all participants; −2.20 (−2.7, −1.7), 0.80 (−1.2, −0.5) and 0.55 (0.1, 1.1) for males; –2.80 (−3.2, −2.5), –1.70 (−2.0, −1.5) and –0.40 (−0.8, 0.2) for females. Chi-squared test was used to check the differences of frequencies in different tertiles of T-score. *p* < 0.05 was considered significant. HDL, high density lipoprotein.

**Table 3:** Odds ratio of full metabolic syndrome and its components at different tertile of T-score (spine) according to sex.

MODEL	ALL				MALES				FEMALES			
	TERTILE			*p*	TERTILE			*p*	TERTILE			*p*
	1	2	3		1	2	3		1	2	3	
	CENTRAL OBESITY											
a	1	1.96 (1.5–2.6)	2.37 (1.8–3.2)	<0.01	1	2.47 (1.3–4.6)	4.07 (2.1–7.9)	<0.01	1.0	1.94 (1.4–2.7)	2.14 (1.5–2.9)	<0.01
b	1	1.95 (1.5–2.6)	2.44 (1.8–2.3)	<0.01	1	2.58 (1.4–4.9)	4.23 (2.2–8.3)	<0.01	1.0	1.95 (1.4–2.7)	2.21 (1.6–3.1)	<0.01
c	1	1.98 (1.4–2.6)	2.24 (1.6–3.1)	<0.01	1	1.94 (0.8–4.6)	3.06 (1.3–7.4)	0.04	1.0	2.01 (1.4–2.8)	2.27 (1.6–3.3)	<0.01
d	1	1.88 (1.4–2.5)	2.21 (1.6–3.0)	<0.01	1	1.97 (0.8–4.7)	2.95 (1.2–7.1)	0.04	1.0	1.82 (1.3–2.5)	1.98 (1.4–2.8)	<0.01
e	1	1.90 (1.4–2.5)	2.20 (1.6–3.0)	<0.01	1	2.00 (0.8–4.8)	3.07 (1.2–7.5)	0.04	1.0	1.83 (1.3–2.6)	1.97 (1.4–2.8)	<0.01
	HYPERGLYCEMIA											
a	1	1.21 (0.9–1.6)	1.23 (0.9–1.6)	0.25	1	0.64 (0.3–1.5)	1.66 (0.6–4.5)	0.12	1.0	1.32 (1.0–1.8)	1.20 (0.9–1.6)	0.16
b	1	1.32 (1.0–1.7)	1.54 (1.2–2.1)	0.01	1	0.82 (0.3–1.9)	2.25 (0.8–6.4)	0.10	1.0	1.40 (1.0–1.9)	1.47 (1.1–2.0)	0.02
c	1	1.28 (1.0–1.8)	1.34 (1.2–2.2)	0.04	1	0.71 (0.3–1.8)	1.71 (0.6–5.1)	0.21	1.0	1.32 (1.0–1.8)	1.44 (1.1–2.0)	0.05
d	1	1.18 (0.9–1.6)	1.29 (0.9–1.7)	0.23	1	0.66 (0.3–1.7)	1.59 (0.5–4.8)	0.24	1.0	1.25 (0.9–1.7)	1.26 (0.9–1.7)	0.29
e	1	1.17 (0.9–1.6)	1.15 (0.8–1.6)	0.57	1	0.62 (0.2–1.7)	1.58 (0.5–5.1)	0.26	1.0	1.25 (0.9–1.7)	1.21 (0.8–1.6)	0.41
	LOW HDL-CHOLESTEROL											
a	1	1.07 (0.8–1.4)	1.28 (1.0–1.6)	0.16	1	1.22 (0.6–2.3)	1.05 (0.6–1.9)	0.80	1.0	1.05 (0.8–1.4)	1.35 (1.0–1.8)	0.09
b	1	1.08 (0.8–1.4)	1.32 (1.0–1.7)	0.11	1	1.43 (0.8–2.7)	1.20 (0.6–2.3)	0.55	1.0	1.05 (0.8–1.4)	1.38 (1.0–1.9)	0.08
c	1	1.05 (0.9–1.6)	1.30 (1.0–1.9)	0.32	1	1.19 (0.6–2.3)	1.01 (0.5–1.9)	0.84	1.0	1.02 (0.8–1.4)	1.29 (0.9–1.8)	0.21
d	1	0.96 (0.7–1.2)	1.13 (0.9–1.5)	0.46	1	1.20 (0.6–2.4)	0.90 (0.4–1.8)	0.69	1.0	0.94 (0.7–1.3)	1.30 (0.9–1.7)	0.21
e	1	0.96 (0.7–1.3)	1.15 (0.9–1.5)	0.39	1	1.17 (0.6–2.4)	0.82 (0.4–1.7)	0.59	1.0	0.94 (0.7–1.3)	1.24 (0.9–1.7)	0.18
	HYPERTRIGLYCERIDEMIA											
a	1	1.27 (1.0–1.6)	1.35 (1.1–1.7)	0.04	1	1.16 (0.6–2.2)	1.51 (0.8–2.8)	0.43	1.0	1.30 (1.0–1.7)	1.34 (1.0–1.7)	0.06
b	1	1.29 (1.0–1.7)	1.42 (1.1–1.8)	0.02	1	1.25 (0.7–2.4)	1.62 (0.8–3.1)	0.34	1.0	1.30 (1.0–1.7)	1.34 (1.0–1.8)	0.07
c	1	1.25 (1.0–1.7)	1.29 (1.2–1.9)	0.04	1	1.18 (0.6–2.3)	1.47 (0.8–2.9)	0.51	1.0	1.20 (0.9–1.6)	1.24 (0.9–1.7)	0.29
d	1	1.18 (0.9–1.5)	1.22 (0.9–1.6)	0.30	1	1.24 (0.6–2.4)	1.38 (0.7–2.7)	0.64	1.0	1.16 (0.9–1.5)	1.13 (0.8–1.5)	0.56
e	1	1.17 (0.9–1.7)	1.15 (0.9–1.5)	0.46	1	1.16 (0.6–2.3)	1.47 (0.7–2.9)	0.56	1.0	1.16 (0.9–1.5)	1.07 (0.8–1.5)	0.61
	HYPERTENSION											
a	1	1.16 (0.9–1.5)	1.27 (1.0–1.6)	0.20	1	0.90 (0.5–1.7)	1.05 (0.6–1.9)	0.88	1.0	1.23 (0.9–1.6)	1.32 (1.0–1.8)	0.15
b	1	1.12 (0.9–1.6)	1.45 (0.9–1.9)	0.06	1	1.07 (0.5–2.1)	1.25 (0.6–2.4)	0.80	1.0	1.27 (0.9–1.7)	1.45 (1.1–1.9)	0.05
c	1	1.20 (0.9–1.7)	1.32 (1.0–1.9)	0.21	1	0.95 (0.5–1.9)	1.10 (0.6–2.2)	0.91	1.0	1.24 (0.9–1.7)	1.37 (1.0–1.9)	0.13
d	1	1.10 (0.8–1.4)	1.24 (0.9–1.6)	0.33	1	0.93 (0.5–1.8)	1.16 (0.6–2.3)	0.79	1.0	1.11 (0.8–1.5)	1.24 (0.9–1.7)	0.40
e	1	1.10 (0.8–1.4)	1.20 (0.9–1.6)	0.50	1	0.90 (0.4–1.8)	1.16 (0.6–2.4)	0.77	1.0	1.11 (0.8–1.5)	1.19 (0.9–1.6)	0.56
	FULL METS											
a	1	1.55 (1.2–2.0)	1.57 (1.2–2.0)	<0.01	1	1.49 (0.8–2.8)	2.46 (1.3–4.7)	0.02	1.0	1.35 (1.0–1.7)	1.45 (1.1–1.9)	<0.01
b	1	1.61 (1.2–2.1)	1.74 (1.3–2.3)	<0.01	1	1.82 (0.9–3.5)	3.05 (1.5–6.0)	<0.01	1.0	1.53 (1.2–2.0)	1.59 (1.2–2.1)	<0.01
c	1	1.54 (1.1–2.0)	1.62 (1.2–2.2)	<0.01	1	1.31 (0.6–2.7)	2.10 (1.1–4.4)	0.03	1.0	1.48 (1.1–2.0)	1.48 (1.1–2.0)	<0.01
e	1	1.59 (1.2–2.1)	1.63 (1.2–2.2)	<0.01	1	1.29 (0.6–2.5)	2.12 (1.1–4.6)	0.03	1.0	1.46 (1.1–2.0)	1.47 (1.1–2.0)	<0.01

Note: Data presented as odds ratio (95% confidence interval) (OR (95% CI)) and respective *p*-values representing odds of having different components of metabolic syndrome at higher tertiles of T-score (spine) compared to the lowest tertile. MetS is full metabolic syndrome; Ref is reference; Ter1, 2 and 3 are different tertiles of T-score. The data was generated by multinomial regression taking T-score tertiles as dependent variables and MetS and its components (present versus absent) as factors. Model “a” is univariate. All other models are additionally adjusted for age (model “b”), BMI (model “c”), all other MetS components (model “d”) and risk factors associated with bone loss like family history of diabetes, osteoporosis, arthritis, whether or not suffering from T2DM, thyroid disease, history of broken bones, etc. (model “e”). For full MetS, model “d” is excluded as it is a combination of these five components. *p *< 0.05 is considered significant.
